# BubbleGun: enumerating bubbles and superbubbles in genome graphs

**DOI:** 10.1093/bioinformatics/btac448

**Published:** 2022-07-07

**Authors:** Fawaz Dabbaghie, Jana Ebler, Tobias Marschall

**Affiliations:** Medical Faculty, Institute for Medical Biometry and Bioinformatics, Heinrich Heine University Düsseldorf, Düsseldorf 40225, Germany; Helmholtz Centre for Infection Research (HZI), Helmholtz Institute for Pharmaceutical Research Saarland (HIPS), Saarbrücken 66123, Germany; Medical Faculty, Institute for Medical Biometry and Bioinformatics, Heinrich Heine University Düsseldorf, Düsseldorf 40225, Germany; Medical Faculty, Institute for Medical Biometry and Bioinformatics, Heinrich Heine University Düsseldorf, Düsseldorf 40225, Germany

## Abstract

**Motivation:**

With the fast development of sequencing technology, accurate *de novo* genome assembly is now possible even for larger genomes. Graph-based representations of genomes arise both as part of the assembly process, but also in the context of *pangenomes* representing a population. In both cases, polymorphic loci lead to *bubble* structures in such graphs. Detecting bubbles is hence an important task when working with genomic variants in the context of genome graphs.

**Results:**

Here, we present a fast general-purpose tool, called BubbleGun, for detecting bubbles and superbubbles in genome graphs. Furthermore, BubbleGun detects and outputs runs of linearly connected bubbles and superbubbles, which we call *bubble chains*. We showcase its utility on de Bruijn graphs and compare our results to vg’s snarl detection. We show that BubbleGun is considerably faster than vg especially in bigger graphs, where it reports all bubbles in less than 30 min on a human sample de Bruijn graph of around 2 million nodes.

**Availability and implementation:**

BubbleGun is available and documented as a Python3 package at https://github.com/fawaz-dabbaghieh/bubble_gun under MIT license.

**Supplementary information:**

Supplementary data are available at *Bioinformatics* online.

## 1 Introduction

*Genome graphs* represent collections of related sequences and have a wide range of applications in various fields of bioinformatics. In *de novo* genome assembly, for instance, graphs are used to represent a universe of plausible genome reconstructions based on a set of input sequencing reads (Miller *et al.*, 2010). Recent developments have enabled even phased assembly (Porubsky *et al.*, 2021), where the maternal and paternal copy of each pair of homologous chromosomes are reconstructed separately. Facilitated by Graphical Fragment Assembly format (GFA) as an exchange data format, modern assembly tools often offer the possibility to export the underlying graphs for downstream applications. Working with graphs directly instead of using ‘flattened’ contigs has been shown to be beneficial, for example, for phased assembly (Garg *et al.*, 2018), but a tool ecosystem to work with these graphs is only slowly emerging.

As a second important application domain, graphs can facilitate a comprehensive representation of genetic variation segregating in a population, called a *pangenome* (Computational Pan-Genomics Consortium, 2018). Such graph-based pangenome representations might replace present reference genomes in the future, emphasizing the need for corresponding tools.

**Fig. 1.**
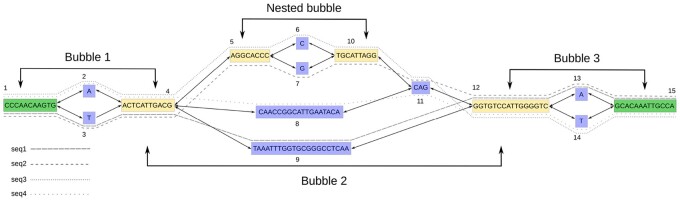
A genome graph resulting from constructing a de Bruijn graph with *k *=* *9 from four sequences (Supplementary Table S2) and subsequently removing overlaps (bluntification). The variances in the sequences give rise to different paths in the graph, constructing a bubble chain of one simple bubble and one superbubble with a simple bubble nested inside

In this work, we focus on bi-directed graphs (Supplementary Section 1.5), where sequences are represented by nodes with a left and right side. Adjacencies are then represented by edges that connect sides of two nodes and can either be non-overlapping (‘blunt’) or represent an overlap between the sequences of the involved nodes. Non-overlapping graphs can arise from pangenomes or from multiple sequence alignments for example (Garrison *et al.*, 2018; Li *et al.*, 2020) and tools for converting from graphs with overlaps to bluntified graphs have been introduced recently (Eizenga *et al.*, 2021). *Bubbles* are key structures within these graphs and can, for example, represent heterozygous variants in assembly graphs or polymorphisms in pangenome graphs. A subgraph between a source node *s* and a sink node *t* is defined as a superbubble (Onodera *et al.*, 2013) if and only if this subgraph is directed, acyclic and the set of nodes reachable from the source *s* is the same set of nodes from where *t* can be reached. Moreover, no other node in the superbubble should satisfy these conditions with either *s* or *t*. A *bubble* then can be defined as a special case of a superbubble, with only two disjoint paths between the source and the sink nodes (Fig. 1). A linear sequence of bubbles is called a *bubble chain*. BubbleGun defines a bubble chain as any linear stretch of one or more bubbles with connected sources and sinks, that is, the sink of one bubble is the source of the next one and so on. The diploid genome assembly method by Garg *et al.* (2018) highlights the importance of bubble chains. In this approach, simple bubbles reflect heterozygous variants. Long reads aligned to paths in the graph can then provide evidence for the haplotype phasing between heterozygous variants encoded in consecutive bubbles. This gives rise to a matrix of (reads times variants) that can be used to compute a bipartition of reads into their respective haplotypes by solving the minimum error correction problem (Lippert *et al.*, 2002).

## 2 BubbleGun

BubbleGun is a fast general purpose tool to detect superbubbles in a given input graph by implementing the algorithm by Onodera *et al.* (2013), which is an average-case linear time algorithm. In a nutshell, the algorithm iterates over all nodes *s* in the graph and determines whether there is another node *t* that satisfies the superbubble rules, a more detailed explanation on the algorithm can be found in Supplementary Section S1.3. Moreover, several attractive features were implemented in BubbleGun that can be useful to the user such as (i) compacting linear stretches of nodes in the graph into one node, (ii) separating the biggest connected component of a graph in terms of node number and (iii) separating a user-specified neighborhood around a node for visualization and investigation purposes. BubbleGun is implemented in Python3 and distributed as Open Source software under the terms of the MIT license. BubbleGun has no extra dependencies other than standard Python3 libraries, which makes it lightweight and easy to integrate in any pipeline without extra overhead or complications from other dependencies.

## 3 Results

### 3.1 Runtime comparison

*Snarls* are a generalized version of superbubbles and BubbleGun was compared with the snarl detection algorithm (Paten *et al.*, 2018) part of the vg toolkit (Garrison *et al.*, 2018). Both tools were tested on two datasets: (i) A de Bruijn graph with a *k*-mer size of 41 representing the pangenome of 10 *Myxococcus xanthus* genomes (Supplementary Table S1) with around 600 000 nodes and (ii) a de Bruijn graph with a *k*-mer size of 61 constructed from short reads from the human sample HG00733 part of the 1000 Genomes Project (1000 Genomes Project Consortium, 2015) with around 22 million nodes. We chose to compare with vg toolkit because: (i) it is a tool widely used in pangenomic research, (ii) it takes GFA files as input which are standard format for genome graphs, (iii) it handles bi-directed graphs and (iv) it reports ‘nestedness’ between bubbles.

The short reads for the HG00733 experiment were first corrected using Lighter (Song *et al.*, 2014). For both datasets, the graphs were constructed using Bcalm2 (Chikhi *et al.*, 2016). The specific *k*-values for both experiments were chosen after building the graphs with several *k*-values in preliminary experiments, where, for each produced graph, we looked at the number of simple bubbles, size of bubble chains and size of the biggest connected component. A *k*-value of 11 for Lighter and 61 for bcalm2 was chosen for the human sample and a *k* value of 41 for the *M.*  *xanthus* experiment. These values lead to a high number of simple bubbles, long bubble chains and graphs that did not fragment into too many components.

Time and memory consumption comparisons showed that for the *M.*  *xanthus* graph, both tools performed relatively similar in terms of running time and memory consumption, with BubbleGun running in 50 s and using 0.56 Gb memory, and VG running in 30 s and using 0.85 Gb memory, where both tools detected 40 381 simple bubbles and 41 356 superbubbles. However, for the HG00733 graph, BubbleGun took around 25 min and used 22 Gb memory, where VG took 67 h and 31 Gb memory with 1 965 000 simple bubbles and 57 089 superbubbles detected by both tools.

### 3.2 Bubble validation

To show the biological importance of bubbles and to validate whether the bubbles detected correspond to true variants instead of repeat collapses or sequencing errors, we used a de Bruijn graph constructed from short reads from the HG002 sample from the Genome in a Bottle (GIAB) consortium (Zook *et al.*, 2016), the *k* size used was 61 as in the previous experiment. We used a GIAB sample in order to take advantage of their high confidence variants to use for the comparison. To generate Variant Call Format (VCF) files from bubble chains, we used a previously established pipeline (Ebler *et al.*, 2022) that detects variants on each complementary path in the bubble chain separately and then merges them into a diploid VCF representation (Supplementary Section S2). Next, using vcfeval (Cleary *et al.*, 2015), we compared the called variants against the high confidence variants from the HG002 sample, looking only at GIAB’s high confidence regions. This resulted in a precision of 95%. As expected, false positive bubbles are enriched in repetitive regions and when excluding regions in the repeat masker track, we observed a precision of 99% and recall of around 97%. False negatives are explained in Supplementary Section S2.1, false positives in Supplementary Section S2.2 and graph preparation steps are explained in more detail in Supplementary Section S2.

## 4 Discussion

We presented BubbleGun, a tool for detecting bubbles, superbubbles and bubble chains in genome graphs. We demonstrated that BubbleGun dramatically reduces the runtime of bubble detection in real-world use cases, paving the way for a more widespread adoption of graph-based workflows. We expect pangenome graphs constructed from *de novo* assemblies to become a broadly used concept where traditional variant detection will be replaced by bubble detection. Diploid genome assembly (Garg *et al.*, 2018) constitutes another important application area. Taken together, we envision BubbleGun to be of broad utility going forward.

## Supplementary Material

btac448_Supplementary_Data

## Data Availability

Human genome Illumina short reads used for HG00733 can be found in SRA with the accession numbers ERR895347, ERR899724, ERR899725, ERR899726, ERR903031 and short read data for HG002/NA24385 can be found under ascending assession numbers ranging from SRR3440404 to SRR3440437. For the M. xanthus experiment, all accession numbers are available in Supplementary Table 1.
