# Supplementary material for: BubbleGun: enumerating bubbles and superbubbles in genome graphs
Source: Bioinformatics. 2022 Jul 7;38(17):4217–9. doi: 10.1093/bioinformatics/btac448 (PMC9438957; doi:10.1093/bioinformatics/btac448)
Supplement: btac448_Supplementary_Data [file btac448_supplementary_data.pdf]

BubbleGun: Enumerating Bubbles and Superbubbles in Genome Graphs. Supplementary material

Fawaz Dabbaghie[1,2], Jana Ebler[1,2], Tobias Marschall[2]

[1] Saarbrücken Graduate School of Computer Science, Saarland University, Saarbrücken, Germany, and [2] Institute for Medical Biometry and Bioinformatics, University Hospital, Heinrich Heine University, Düsseldorf, 40225, Germany.

June 15, 2022

**Assembly Accession**	Bioproject	Biosample	Tax id	Species Tax id	Organism name	Infraspecific name	Version status	Assembly level	Release Type	Genome rep
GCA_000012685.1	PRJNA1421	SAMN02604018	246197	34	Myxococcus xanthus	strain=DK 1622	latest	Complete Genome	Major	Full
GCA_000278585.2	PRJNA168264	SAMN02471831	1198133	34	Myxococcus xanthus	strain=DZ2	latest	Contig	Major	Full
GCA_000340515.1	PRJNA168421	SAMN02471832	1198538	34	Myxococcus xanthus	strain=DZF1	latest	Contig	Major	Full
GCA_006400955.1	PRJNA342411	SAMN05757018	34	34	Myxococcus xanthus	strain=GH3.5.6c2	latest	Complete Genome	Major	Full
GCA_006401215.1	PRJNA342411	SAMN05757019	34	34	Myxococcus xanthus	strain=GH5.1.9c20	latest	Complete Genome	Major	Full
GCA_006401635.1	PRJNA342411	SAMN05757020	34	34	Myxococcus xanthus	strain=KF3.2.8c11	latest	Complete Genome	Major	Full
GCA_006402015.1	PRJNA342411	SAMN05757021	34	34	Myxococcus xanthus	strain=KF4.3.9c1	latest	Complete Genome	Major	Full
GCA_006402415.1	PRJNA342411	SAMN05757022	34	34	Myxococcus xanthus	strain=MC3.3.5c16	latest	Complete Genome	Major	Full
GCA_006402735.1	PRJNA342411	SAMN05757023	34	34	Myxococcus xanthus	strain=MC3.5.9c15	latest	Complete Genome	Major	Full
GCA_900106535.1	PRJEB16561	SAMN05444383	34	34	Myxococcus xanthus	strain=DSM 16526	latest	Scaffold	Major	Full

**Assembly Accession**	Seq Release Date	Assembly name	Submitter	Paired assembly comparison	FTP path
GCA_000012685.1	2006/06/07	ASM1268v1	TIGR	identical	ftp://ftp.ncbi.nlm.nih.gov/genomes/all/GCA/000/012/685/GCA_000012685.1_ASM1268v1
GCA_000278585.2	2013/02/10	ASM27858v2	University of Iowa	identical	urlftp://ftp.ncbi.nlm.nih.gov/genomes/all/GCA/000/278/585/GCA_000278585.2_ASM27858v2
GCA_000340515.1	2013/02/13	Myxococcus xanthus DZF1	University of Iowa	identical	urlftp://ftp.ncbi.nlm.nih.gov/genomes/all/GCA/000/340/515/GCA_000340515.1_Myxococcus_xanthus_DZF1
GCA_006400955.1	2019/06/24	ASM640095v1	ETH Zurich	identical	urlftp://ftp.ncbi.nlm.nih.gov/genomes/all/GCA/006/400/955/GCA_006400955.1_ASM640095v1
GCA_006401215.1	2019/06/24	ASM640121v1	ETH Zurich	identical	ftp://ftp.ncbi.nlm.nih.gov/genomes/all/GCA/006/401/215/GCA_006401215.1_ASM640121v1
GCA_006401635.1	2019/06/24	ASM640163v1	ETH Zurich	identical	ftp://ftp.ncbi.nlm.nih.gov/genomes/all/GCA/006/401/635/GCA_006401635.1_ASM640163v1
GCA_006402015.1	2019/06/24	ASM640201v1	ETH Zurich	identical	urlftp://ftp.ncbi.nlm.nih.gov/genomes/all/GCA/006/402/015/GCA_006402015.1_ASM640201v1
GCA_006402415.1	2019/06/24	ASM640241v1	ETH Zurich	identical	urlftp://ftp.ncbi.nlm.nih.gov/genomes/all/GCA/006/402/415/GCA_006402415.1_ASM640241v1
GCA_006402735.1	2019/06/24	ASM640273v1	ETH Zurich	identical	urlftp://ftp.ncbi.nlm.nih.gov/genomes/all/GCA/006/402/735/GCA_006402735.1_ASM640273v1
GCA_900106535.1	2016/10/22	IMG-taxon 2693429903	DOE	identical	urlftp://ftp.ncbi.nlm.nih.gov/genomes/all/GCA/900/106/535/GCA_900106535.1_IMG-taxon_2693429903_annotated_assembly

Supplementary Table. 1: Information on the 10 *Myxococcus xanthus* used for testing and results

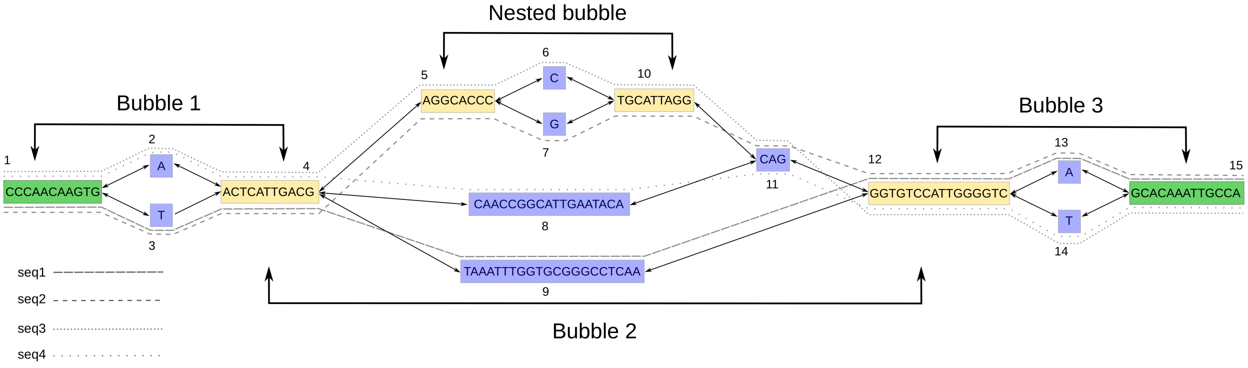

Supplementary Figure. 1: Bluntified de Bruijn graph constructed from 4 sequences presented in Supplementary Table

1 More on methods

1.1 Building de Bruijn Graphs

For the HG00733 experiment the following steps were taken to construct the graphs. First of all, short reads were corrected using `Lighter` (Song *et al.*, 2014). Afterwards, to choose the k-mer size, we simply built the de Bruijn graph with different k values and checked which value seems to produce the least disconnected graph with the most simple bubbles, and longest bubble chains, where we found that a value of $k = 61$ is a good value for this dataset. To build the de Bruijn graph, `bcalm2` (Chikhi *et al.*, 2016) was used on the corrected reads. For the *Myxococcus xanthus* dataset, the assemblies were given to `bcalm2` to produce a de Briujn graph, we also used several k values and found that $k = 41$ gives the least disconnected graph with a high value of simple bubbles and longest bubble chains.

1.2 Building a pangenome

To test `BubbleGun` against pangenomes. We took the 10 *M. xanthus* assemblies and built a pangenome using `minigraph2` (Li *et al.*, 2020). This produces a bluntified (no overlaps) graph with 3,775 nodes and 5,043 edges. `BubbleGun` was able to detect 655 simple bubbles, 314 superbubbles, and 75 insertions, which are simple bubbles but one of the branches does not have a node, and the bubble chains detected covered almost 99% of the sequences in this pangenome graph

1.3 Algorithm

The algorithm presented by Onodera *et al.* (2013) keeps a dynamic set S that contains the nodes that are possible to visit in the next iterations, once one of

Sequence_1	CCCAACAAGTGTACTCATTGACGTAAATTTGGTGCGGGCCTCAAGGTGTCCATTGGGGTCAGCACAAATTGCCA
Sequence_2	CCCAACAAGTGTACTCATTGACGAGGCACCCGTGCATTAGGCAGGGTGTCCATTGGGGTCTGCACAAATTGCCA
Sequence_3	CCCAACAAGTGAACTCATTGACGAGGCACCCCTGCATTAGGCAGGGTGTCCATTGGGGTCAGCACAAATTGCCA
Sequence_4	CCCAACAAGTGAACTCATTGACGCAACCGGCATTGAATACACAGGGTGTCCATTGGGGTCTGCACAAATTGCCA

Supplementary Table. 2: Sequences used to generate the example in Supplementary Figure 1 and Figure 1

these node is visited, it is popped from S. It also keeps track of *visited* and *seen* nodes, where seen nodes are nodes that have at least one visited parent. The algorithm aborts when finding a node with no children (a tip) or an edge that points back to s (a cycle). Once there is only one node t left in S and no other nodes are marked as seen the algorithm then returns t as the sink.
As mentioned in the original publication of the algorithm, it has an average-case linear time complexity of $\mathcal{O}(n + m)$. The algorithm is in practice fast as it aborts when it hits a cycle or a tip. Therefore, when it starts at each node in the graph, it will either find a bubble quickly as simple bubbles are small, a superbubble, or aborts from encountering a tip or a cycle. A very big superbubble could be large in size, causing a longer time to catch. However, in case of a big superbubbles, once its found, all its nodes are marked as visited, so the algorithm will not look at these nodes again. From the results, we see that it scales rather good, even on a human-size de Bruijn graph, it finished in a very reasonable time.
In Algorithm 1 describes the algorithm used in `BubbleGun`, it is the same algorithm in Onodera *et al.* (2013), with a small modification of adding the directionality. With this modification, we do not need to have a directed graph as input, but we have a bidirected graph. However, for every node v in the graph, we look at both direction, and from that point, every time we enter a child node from one side, we exit from the other side during the traversals. For example, if we a node v have two children u_1 and u_2 from the right side, and the two edges from the right side of v enter both u_1 and u_2 from their left side, then when we at a later time pop u_1 or u_2, we have to continue our traversal and look at their children from the right side and not go back to v from their left side.

1.4 Nested bubbles

As the algorithm goes through each node and start looking for a bubble or a superbubble, we do not know beforehand whether this bubble is nested or not. Therefore, after detecting all bubbles and superbubbles the algorithm would be called recursively on each superbubble, if there were any structures nested inside this superbubble, they will be reported and marked with their correct parent superbubble id and parent chain id. This information can be then found in the JSON output which can be easily parsed and used in any downstream analyses needed by the user. We found in practice that these nested calls do not take much time.

Algorithm 1 BubbleGun detection algorithm

Input: Bidirected graph $G(V, E)$

```
for v ∈ V do
   for direction in {0, 1} do
      push v into S
      while |S| ≠ 0 do
         pop some node n from S
         mark n as visited
         if n does not have children in direction then
            abort {it is a tip}
         end if
         for u ∈ n's children in direction do
            if u == n then
               abort {we have a cycle pointing back to n}
            end if
            mark u as seen
            if all of u's parents are visited then
               push u into S
            end if
         end for
         if one node t is left in S and no other nodes are seen then
            if no edge between n and t then
               return  t {we found the sink}
            else
               abort {we have a cycle with s}
            end if
         end if
      end while
   end for
end for
```

1.5 Bidirected Graphs

Formally defined, a bi-directed graph is a graph $D = (V_D \times \{left, right\}, E_D)$, where each node $v \in V_D$ has two "sides", a left and a right side. The underlying idea being that one "enters" a node v from one side and "leaves" it through the other side. Edges then connect a side of one node to a side of another node, or can connect two sides of the same nodes in case of a loop or a repeat for example (Paten *et al.*, 2018).
Therefore, A genomic bi-directed graph is a special graph where the direction of the traversal indicates whether we are reading the forward or the reverse complement of the sequence in the vertex. In the Figure 2, we see that each node represents a sequence, and there is a red side (left) and a yellow side (right), the red traversal will spell ATGTCCGGC and it starts from node 1 at the red side and exits the yellow, then enters the red side on node 2 and exits the yellow and so on, if a traversal enters a node from "start" (red) and exits from "end" (yellow) then we read the forward sequence, if a traversal enters from "end" (yellow) and exits from "start" (start), then we read the reverse complement of that sequence in that node. We can see that the green traversal starts from the yellow on 4 and exits from red at 1 and spells the sequence GCCAATCAT.
One does not need to index both sequence and its reverse complement, but one can either keeps the forward or the reversed, and depending on the traversal direction the string in the vertex is taken as is or reversed. If for example, node 1 yellow side was connected with node 2 yellow, then the sequence will be ATGGGA (because we entered from the yellow side of node 2, we need to read the reverse complement of that sequence in 2).
`BubbleGun` keeps track of this directionality and when traversing, it makes sure that when we enter from one side, we have to exit from the next and not go back by mistake.

2 Bubbles Validation

For the bubbles validation experiment, we used the HG002 sample from Genome In A Bottle Consortium (GIAB) (Zook *et al.*, 2016). First step, we downloaded the Illumina paired-end short reads and used `lighter` (Song *et al.*, 2014) to correct them. Second step was to construct a de Bruijn graph from the corrected reads using `bcalm2`. Tips from the graph were removed using the `UntipRelative` script from `GraphAligner` (Rautiainen and Marschall, 2019), then the graph was compacted using `BubbleGun compact` subcommand and simple bubble chains were detected. To find out if a bubble represent a variant in the genome, and where in the genome this variant is located, we extracted two complementary sequences from every simple bubble chain, where a sequence is the concatenation of the node labels - with overlaps removed - of a path going from one end of the chain to the other. The two sequence represent two randomly-chosen haplotypes. Supplementary Figure 3 is an example of a bubble chains made of 3 simple bubbles, one can take two complementary paths

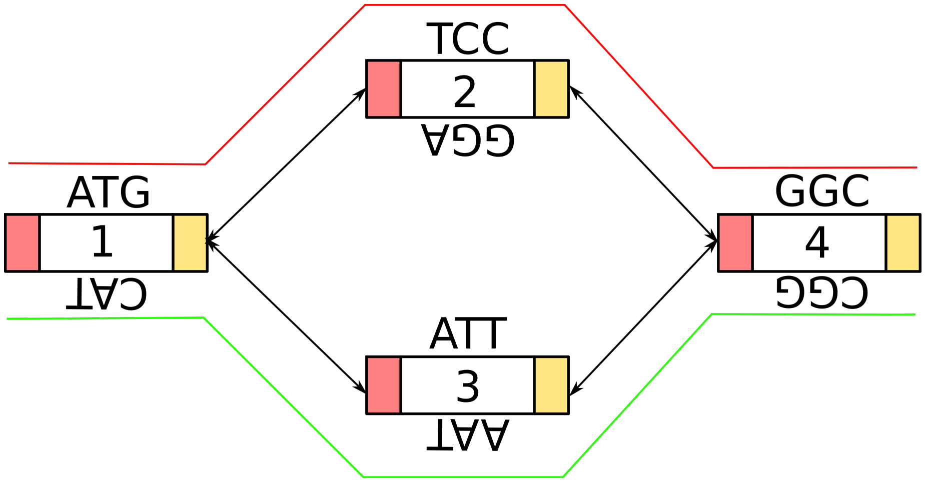

Supplementary Figure. 2: An example of a bidirected graph, where nodes have a left (red) and right (yellow) sides. And an edge connectes two side together.

through this bubble chains where bubble branches are marked randomly with 0 or 1, then one path can be 1-0-1 and the other 0-1-0 for example, both paths will go through the shared sources and sinks. Therefore, we can obtain two sequences from a bubble chain with simple bubbles.

We then aligned these haplotypes back to the reference genome using `minimap2` (Li, 2018), called variants separately on each haplotype using the `paftools.js` script with the `minimap2` package and then merged the variants into a diploid VCF representation, using a similar pipeline to call variants presented in (Ebler *et al.*, 2020). In the merging step, sets of variant alleles overlapping across bubble chains are represented as a single variant record in the VCF with multiple alternative alleles reflecting all allele sequences observed in the respective genomic region. Supplementary Figure 4 provides an illustration: on top, variant alleles called from the alignments of bubble chains to the reference are shown together with their VCF representation. Below, we show how the resulting variant records after merging. As the rightmost deletion overlaps with the SNP and the insertion called from the first bubble chain, the locus is represented in terms of a single variant record with two alternative allele sequences in the merged VCF.

Commands used are all present and explained in the `BubbleGun` Github repository in the "Use Cases" folder. The pipeline used for variant calling the bubbles can be found here `https://bitbucket.org/jana_ebler/vcf-merging/src/chains-genotyping/`
After producing a VCF file from the aforementioned pipeline, we compared our variants with the high-confidence variants produced for the HG002 sample in

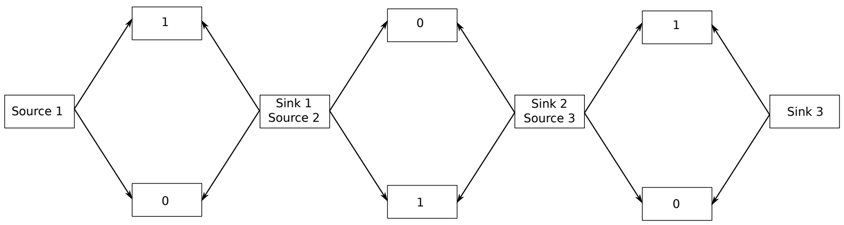

Supplementary Figure. 3: An example of a simple bubble chains with 3 bubbles, the branches of each bubble are assigned either 0 or 1 randomly. Two complementary walks can be extracted from this chain

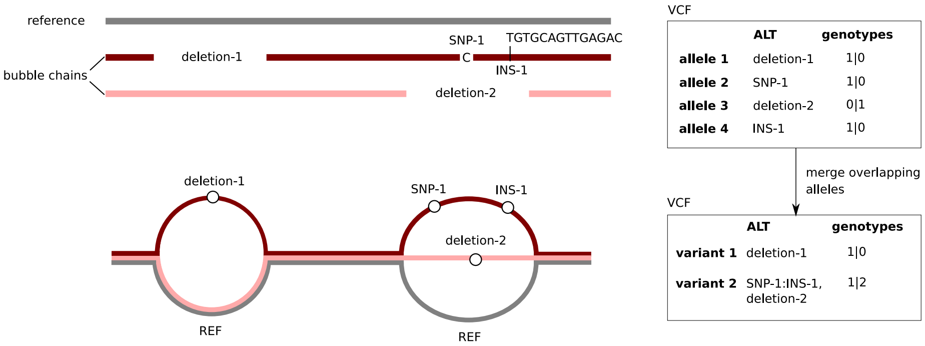

Supplementary Figure. 4: **Allele merging.** When calling variants from bubble chains, sets of overlapping alleles are merged to form a single, multi-allelic variant record.

the Genome in Bottle Project, we used RTG Tools `vcfeval` for this (Cleary *et al.*, 2015). We found that 5% were false positive, after filtering against repeat regions using a BED file of the repeat masker track from the Genome Browser, we got a percision of around 99%, and a recall of around 97%. Therefore, having around 1% of False Positives and around 3% of False Negatives.

2.1 False Negatives Assessment

After removing the repeats from the variants detected from the simple bubbles chains from the HG002 sample, we got a recall of around 97%. Which means there is a 3% of False Negatives. This can be explained by the superbubble structures, as we only separated two complementary paths from bubble chains made of simple bubbles, we did not include superbubbles, as it is a harder problem to extract two disjoint (complementary paths) from a superbubble.

To show this, we extracted some flanking regions from the human genome reference with size 1000 bp, where we take 500 bp before and after a false negative on the condition that also this flanking region contains a True Positive. We mapped these regions back on the de Bruijn graph produced from the short reads of the HG002 sample to see why would we have a False Negative, we used `GraphAligner` (Rautiainen and Marschall, 2019), to map these sequences back to the graph.
Figure 5 shows 3 examples of these aligned sequences back to graph, we used `Bandage` for visualization, and `BubbleGun bfs` subcommand to separate a small neighborhood in the graph to visualize, as the graph is very big and cannot be visualized in its entirety. We can see that these are chains of simple bubbles and superbubbles, and the nodes with blue borders represent the path our region took in the graph, and due to the presence of superbubbles that we did not include in our bubbles validation, these have caused the False Negatives.

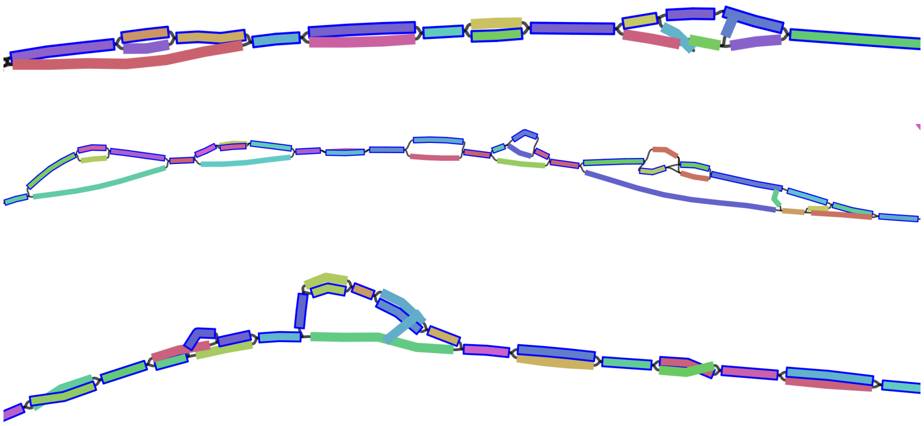

Supplementary Figure. 5: Three examples of bubbles chains containing simple and superbubbles, the nodes with blue borders represent the path the sequence we separated from the reference mapped to. These regions were separated using `BubbleGun bfs` subcommand which allows the user to give one or more start node and a neighborhood size, `BubbleGun` then produces a new GFA file with the nodes around the start node of the size the user specified, this help in visualizing small parts of big graphs

2.2 False Positives Assessment

Potential reasons of having around 1% of False Positives:

1. Positions in the genome where 3 or more raw reads had the same error. As `bcalm2` was set to an abundance minimum of 3, which means that if

the k-mer appears in 3 reads or more, it will be included in the graph. Supplementary Figure 6

2. Alignment problems towards the end of a haplotype alignment. This can also be seen when aligning long reads sometimes. Supplementary Figure 7 is an example.

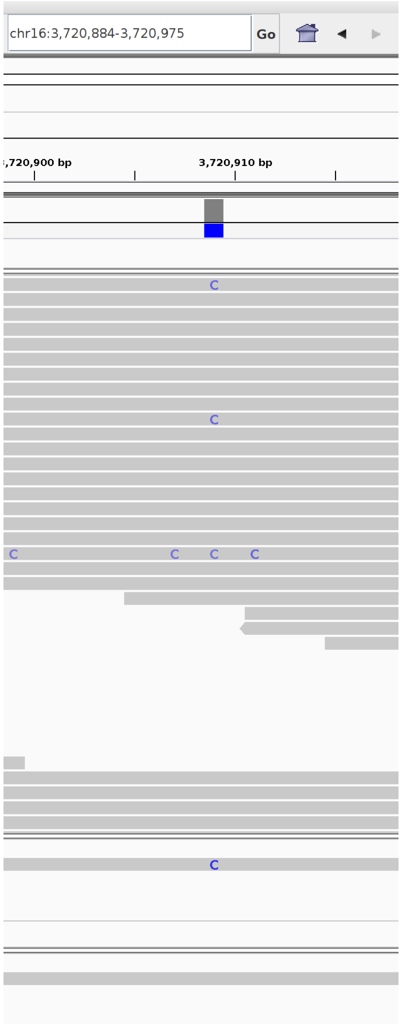

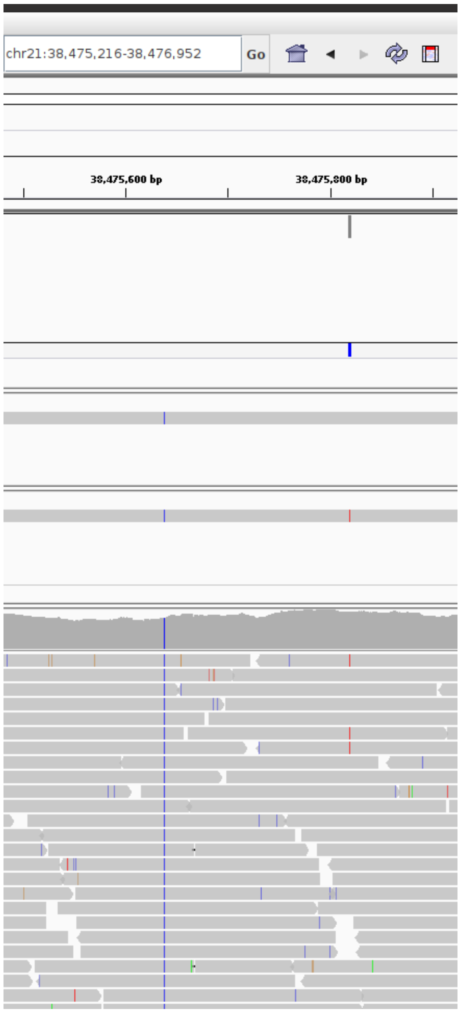

Supplementary Figure. 6: Two screenshots from IGV (Interactive Genome Viewer) showing the false positive variant and that 3 raw reads containing that variant at that position

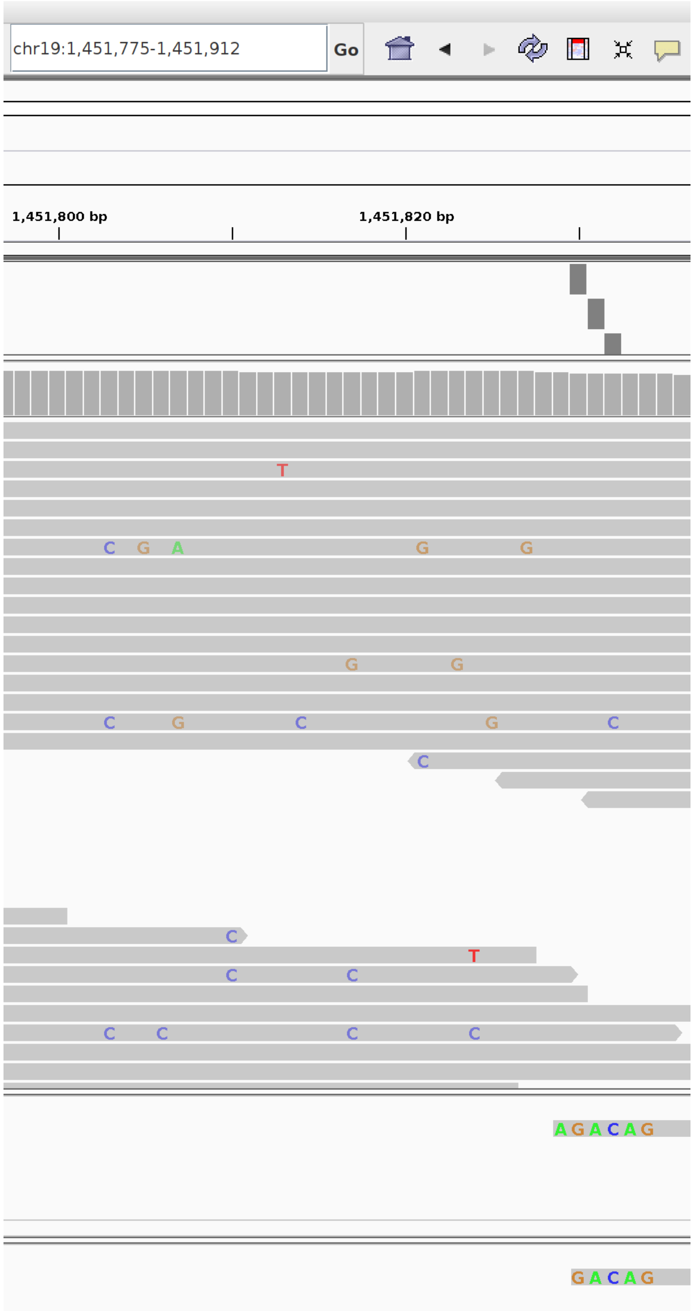

Supplementary Figure. 7: The two bottom tracks are the two haplotypes aligned, and we see that the first 5-6 amino acids of the haplotypes are erroneous, and could be caused because of the highly erroneous short reads in this section of the alignment

References

Chikhi, R. *et al.* (2016). Compacting de bruijn graphs from sequencing data quickly and in low memory. *Bioinformatics*, **32**(12), i201–i208.

Cleary, J. G. *et al.* (2015). Comparing variant call files for performance benchmarking of Next-Generation sequencing variant calling pipelines.

Ebler, J. *et al.* (2020). Pangenome-based genome inference.

Li, H. (2018). Minimap2: pairwise alignment for nucleotide sequences. *Bioinformatics*, **34**(18), 3094–3100.

Li, H. *et al.* (2020). The design and construction of reference pangenome graphs with minigraph. *Genome Biol.*, **21**(1), 265.

Onodera, T. *et al.* (2013). Detecting superbubbles in assembly graphs. In *Algorithms in Bioinformatics*, pages 338–348. Springer Berlin Heidelberg.

Paten, B. *et al.* (2018). Superbubbles, ultrabubbles, and cacti. *J. Comput. Biol.*, **25**(7), 649–663.

Rautiainen, M. and Marschall, T. (2019). GraphAligner: Rapid and versatile Sequence-to-Graph alignment.

Song, L. *et al.* (2014). Lighter: fast and memory-efficient sequencing error correction without counting. *Genome Biol.*, **15**(11), 509.

Zook, J. M. *et al.* (2016). Extensive sequencing of seven human genomes to characterize benchmark reference materials. *Sci Data*, **3**, 160025.
